# Activin A regulates the epidermal growth factor receptor promoter by activating the PI3K/SP1 pathway in oral squamous cell carcinoma cells

**DOI:** 10.1038/s41598-019-41396-7

**Published:** 2019-03-26

**Authors:** Chi-Neu Tsai, Chia-Lung Tsai, Jui-Shan Yi, Huang-Kai Kao, Yenlin Huang, Chun-I Wang, Yun-Shien Lee, Kai-Ping Chang

**Affiliations:** 1 Graduate Institute of Clinical Medical Sciences, Chang-Gung University, Guishan Dist., Taoyuan City, 33302 Taiwan; 2 Department of Surgery, Chang-Gung Memorial Hospital, Guishan Dist., Taoyuan City, 33305 Taiwan; 3 Genomic Medicine Core Laboratory, Chang Gung Memorial Hospital, Guishan Dist., Taoyuan City, 33305 Taiwan; 4 Department of Otolaryngology-Head & Neck Surgery, Chang Gung Memorial Hospital, Guishan Dist., Taoyuan City, 33305 Taiwan; 5 Molecular Medicine Research Center, Chang Gung University, Guishan Dist., Taoyuan City, 33302 Taiwan; 6 Department of Plastic & Reconstructive Surgery, Chang Gung Memorial Hospital, Guishan Dist., Taoyuan City, 33305 Taiwan; 7 Department of Pathology, Chang Gung Memorial Hospital, Guishan Dist., Taoyuan City, 33305 Taiwan; 8 Department of Biotechnology, Ming-Chuan University, Guishan Dist., Taoyuan City, 33348 Taiwan

## Abstract

Epidermal growth factor receptor (EGFR) and activin A are both overexpressed in oral cavity squamous cell carcinoma (OSCC). We evaluated their clinical correlation and activin A-mediated EGFR regulation in this study. Overexpression of both transcripts/proteins indicated a poorer prognosis in OSCC patients. Knockdown of endogenous *INHBA* repressed the expression of EGFR and inhibited activin A-mediated canonical Smads, noncanonical phosphorylation of AKT (ser473) (p-AKT ser473) and SP1. Inhibition of PI3K signaling via its inhibitor attenuated p-AKT ser473 and in turn reduced SP1 and EGFR expression in the presence of recombinant activin A (rActivin A) in OSCC cells, as revealed via a luciferase assay and western blotting. However, canonical Smad signaling repressed the EGFR promoter, as revealed by a luciferase assay. The transcription factor SP1, its coactivator CBP/p300, and Smad proteins were recruited to the EGFR proximal promoter following rActivin A treatment, as revealed by chromatin immunoprecipitation (ChIP). Smad2/3/4 dramatically outcompeted SP1 binding to the EGFR proximal promoter following mithramycin A treatment. Activin A activates the PI3K and Smad pathways to compete for binding to overlapping SP1 consensus sequences on the EGFR proximal promoter. Nevertheless, canonical p-Smad2 was largely repressed in OSCC tumor tissues, suggesting that the activin A-mediated noncanonical pathway is essential for the carcinogenesis of OSCC.

## Introduction

Oral cavity cancer is among the most common cancers worldwide, accounting for approximately 11,000 deaths per year^1^. Squamous cell carcinoma (SCC) is the most common among a variety of oral cavity cancers and can be found in various locations, including the tongue, gingiva lips, buccal cavity, mouth floor and hard palate^2^. Despite recent advances in surgical, radiotherapy, and chemotherapy treatment protocols, the five-year survival rate of patients remains approximately 60%^3,4^. Most treatment failures occur due to local-regional recurrence or distant metastasis^3,4^. Therefore, clarifying the molecular tumorigenesis mechanisms of oral cavity squamous cell carcinoma (OSCC) tumors is still challenging for the development of new treatment strategies.

Activin A, which is encoded by the *INHBA* gene, is a secreted molecule belonging to the transforming growth factor β (TGF-β) family that mediates various cellular activities and cancer progression^5–7^. Canonical TGF-β signaling triggered by the binding of ligands to its type II receptor results in the recruitment, phosphorylation and subsequent activation of the type I receptor. The phosphorylated type I receptor phosphorylates a subset of receptor-regulated Smad proteins (R-Smads; Smad2, Smad3), which translocate into the nucleus and directly bind regulatory promoters or form complexes with common-Smad (Co-Smad; Smad4), a component of the postreceptor signal transduction system^8^. In addition to the canonical pathway, TGF-β activates the c-Jun N-terminal kinase (JNK), p38 mitogen-activated protein kinase (MAPK), NF-*k*B and phosphatidylinositol 3′-kinase (PI3K)–protein kinase B (AKT) pathways via a noncanonical pathway that is considered essential for cancer progression, inflammatory responses, and developmental cues^9–11^. Activin A is overexpressed or dysregulated in various cancers, including colon, oral, esophageal, ovarian, prostate, pancreatic, and lung cancers; however, the biological effects of activin A on cancer cell progression remain controversial^12–17^. According to a review of the literature, activin A has been reported to activate canonical Smad pathways to regulate biological processes^15,18,19^. Beyond the Smad pathways, it is still unclear if the abovementioned TGF-β-induced noncanonical pathways are also stimulated via activin A in cancers.

EGFR is a member of a superfamily of ErbB tyrosine kinases that trigger downstream signaling cascades to promote cell proliferation and migration^20^. Therefore, EGFR is an important oncological target with effective therapeutics for several cancers^20,21^. The *EGFR* promoter is TATA-less and GC-rich, and multiple transcriptional initiations have been reported; therefore, the +1 of the *EGFR* promoter has been used frequently for convenient translational initiation (Fig. S1)^22^. Based on previous reports, the region approximately ~500 bp upstream of the translation initiation site in *EGFR*, possesses essential promoter activity^23,24^, which can be modulated via SP1, activator protein 1 (AP1), P53, Yin Yang 1 (YY1), WT1, early growth response-1 (EGR-1), and TGFβ-inducible early gene-1 (TIEG1)(Fig. S1)^23,25–28^. Among these response elements are two core SP1 binding consensus sites, i.e., [5′-(G/T)GGGCGG(G/A)(G/A)(C/T)-3′] (site I: -108 to -103; site II: -144 to -139), located on the *EGFR* proximal promoter, which has been reported to be crucial for its basal activity; furthermore, the interaction between SP1 and other transcription factors is essential for modulation of its expression^23,27,29^.

Previously, activin A has been reported to activate the DNA-binding and transactivation potential of SP1 to stimulate *vascular endothelial growth factor* (*VEGF*) gene transcription in human hepatocellular carcinoma cells via unknown mechanisms^30^. Therefore, we presumed that *EGFR* should be an activin A target gene through SP1 activation; however, the regulation of activin A and *EGFR* has never been reported, at least in oral cancer cells. Furthermore, a previously unreported potential Smad binding element (SBE, CAGA, -139 to -136)^31^ overlapped with the site II SP1 consensus sequences in the *EGFR* proximal promoter, but the interaction between SP1 and Smads is also unclear. Therefore, in this study, we aimed to elucidate the regulatory mechanism underlying activin A-mediated EGFR expression; the interactions among activin A stimulation, SP1 and canonical Smads in EGFR transcript/expression; and the clinical correlation of activin A versus EGFR in OSCC cells.

## Results

### Clinical correlation of activin A and EGFR in tumor cells from OSCC tissues

**Figure 1:**
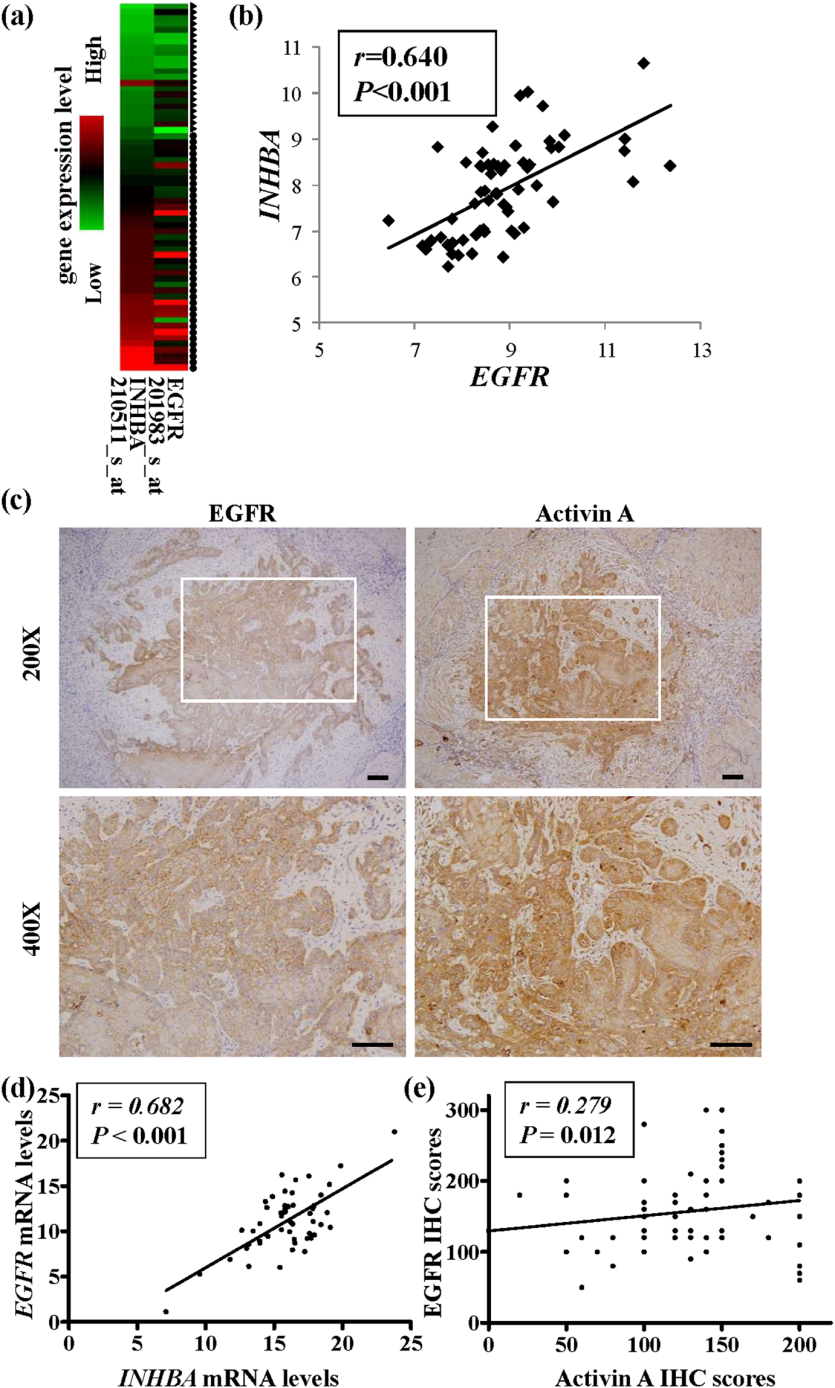
Overexpression and correlation of activin A and EGFR in OSCC tissues. (**a**) The correlation of *INHBA* expression and *EGFR* expression in OSCC tissues was analyzed using Affymetrix U133A chip data. Transcripts of *INHBA* and *EGFR* in normal (*n* = 22) and OSCC tissues (*n* = 40) were analyzed using data from Affymetrix U133A chips. Total RNA was isolated from each OSCC and normal sample. Triangle: normal tissue; Circle: OSCC tissue. The probe set numbers of these two genes are shown on the X-axis; gene expression levels of each sample are shown on the Y-axis. Red indicates high transcript expression; green indicates low expression. (**b**) The correlation between *INHBA* and *EGFR* transcripts in OSCC tissues (*n* = 40) was analyzed by performing Pearson’s correlation analysis. Each squared spot indicates array (chip) data for a patient, and the X-axis and Y-axis represent the log2 ratio value of *EGFR* and *INHBA* on the chip, respectively. (**c**) Immunohistochemical staining of activin A and EGFR in OSCC tumor tissues from one representative case (scale bar: 100 μm). Expression (brown staining) of activin A and EGFR indicates that these proteins localized in the membrane or cytoplasm of OSCC tumor cells. Images shown in the box (upper panel, 200X) were enlarged and are shown in the lower panel (400X). (**d**) Pearson’s correlation analysis of mRNA expression levels of *EGFR* and *INHBA* in 50 pairs of OSCC tumor versus normal tissues. (**e**) Pearson’s correlation of immunohistochemical scores of EGFR and activin A in 155 enrolled OSCC specimens. Expression was considered significant when *P* < 0.05.

The clinical correlation between activin A and EGFR was addressed in clinical OSCC specimens. At first, a correlation was observed between the transcripts of *EGFR* and those of *INHBA* in OSCC tissues (*n* = 40) by analyzing in our microarray data (Fig. 1a), and the correlation between these two genes was significant according to a Pearson’s correlation analysis (*r* = 0.640, *P* < 0.001) (Fig. 1b). To identify the cell types in the tumor mass that expressed both activin A and EGFR, we performed immunohistochemical staining of tissue sections from 155 patients with OSCC. Activin A and EGFR were highly expressed in the cytoplasm and membrane of the tumor cells (Fig. 1c). The *INHBA* and *EGFR* mRNA levels in the OSCC tissues were significantly correlated (*r* = 0.682, *P* < 0.001; Fig. 1d). Similarly, the correlation between the two proteins’ immunohistochemical scores was statistically significant (*r* = 0.279, *P* = 0.012; Fig. 1e).

**Figure 2:**
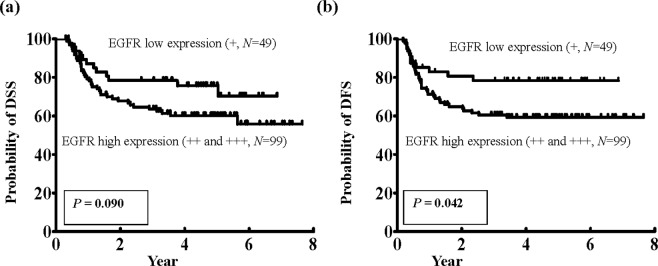
Association between high EGFR expression in the immunohistochemistry analysis and poor prognoses of patient disease-specific survival (DSS) and disease-free survival (DFS). (**a**) Kaplan-Meier plot showing that the 5-year DSS rates in patient subgroups stratified by EGFR expression levels were 74.8% versus 60.1% (*P* = 0.090). (**b**) Kaplan-Meier plot showing that the 5-year DFS rates in patient subgroups stratified by EGFR expression levels were 73.8% versus 59.3% (*P* = 0.042).

The expression levels of EGFR in the immunohistochemistry analysis were significantly correlated with a positive pN status (*P* = 0.030), overall pathological stage (*P* = 0.041), perineural invasion (*P* = 0.038), and tumor depth (*P* = 0.037) (Table 1). Based on a survival analysis of 148 patients undergoing complete standardized treatment and regular follow-up, the five-year disease-specific survival (DSS) rates in the patient subgroups stratified by low and high expression levels of EGFR were 74.8% and 60.1%, respectively, but this difference was not statistically significant using a log-rank test (*P* = 0.09) (Fig. 2a). However, in a comparison of the long-term disease-free survival (DFS) in the patient subgroups stratified by low and high expression levels of EGFR, the five-year DFS rates were 73.8% and 59.3%, respectively (*P* = 0.042) (Fig. 2b). These findings indicated the presence of an association between activin A and EGFR; the high expression of EGFR was also associated with poor prognositc factors and posttreatment survival of OSCC patients.

**Table 1:** Association of EGFR expression levels (immunohistochemical score) with clinicopathological characteristics in 155 untreated OSCC patients.

	No.	EGFR	*p*-value
		Immunohistochemical score	
**Gender**			
Female	15	124 ± 50	0.504
Male	140	158 ± 53	
**Age**			
<50	78	149 ± 51	0.228
>50	77	159 ± 57	
**pT Status**			
1–2	84	149 ± 52	0.351
3–4	71	159 ± 56	
**pN Status**			
(−)	100	149 ± 57	0.030^†^
(+)	55	163 ± 47	
**Overall Pathological Stage**			
I–II	54	144 ± 56	0.041^†^
III–IV	101	160 ± 52	
**Cell differentiation** ^*^			
W-D + M-D	141	152 ± 54	0.060
P-D	14	178 ± 49	
**Perineural Invasion**			
No	108	148 ± 55	0.038^†^
Yes	47	167 ± 49	
**Tumor depth** (mm)			
<=8	80	146 ± 55	0.037^†^
>8	75	163 ± 52	

^*^W-D: well-differentiated, M-D: moderately differentiated, and P-D: poorly differentiated squamous cell carcinoma.

^†^Statistically significant.

### Endogenous expression of phospho-Smad2 (p-Smad2) ser465/467, phospho-AKT (p-AKT) ser473, SP1 and EGFR was repressed in OC3 cells expressing *INHBA*-specific RNA interference (RNAi)

**Figure 3:**
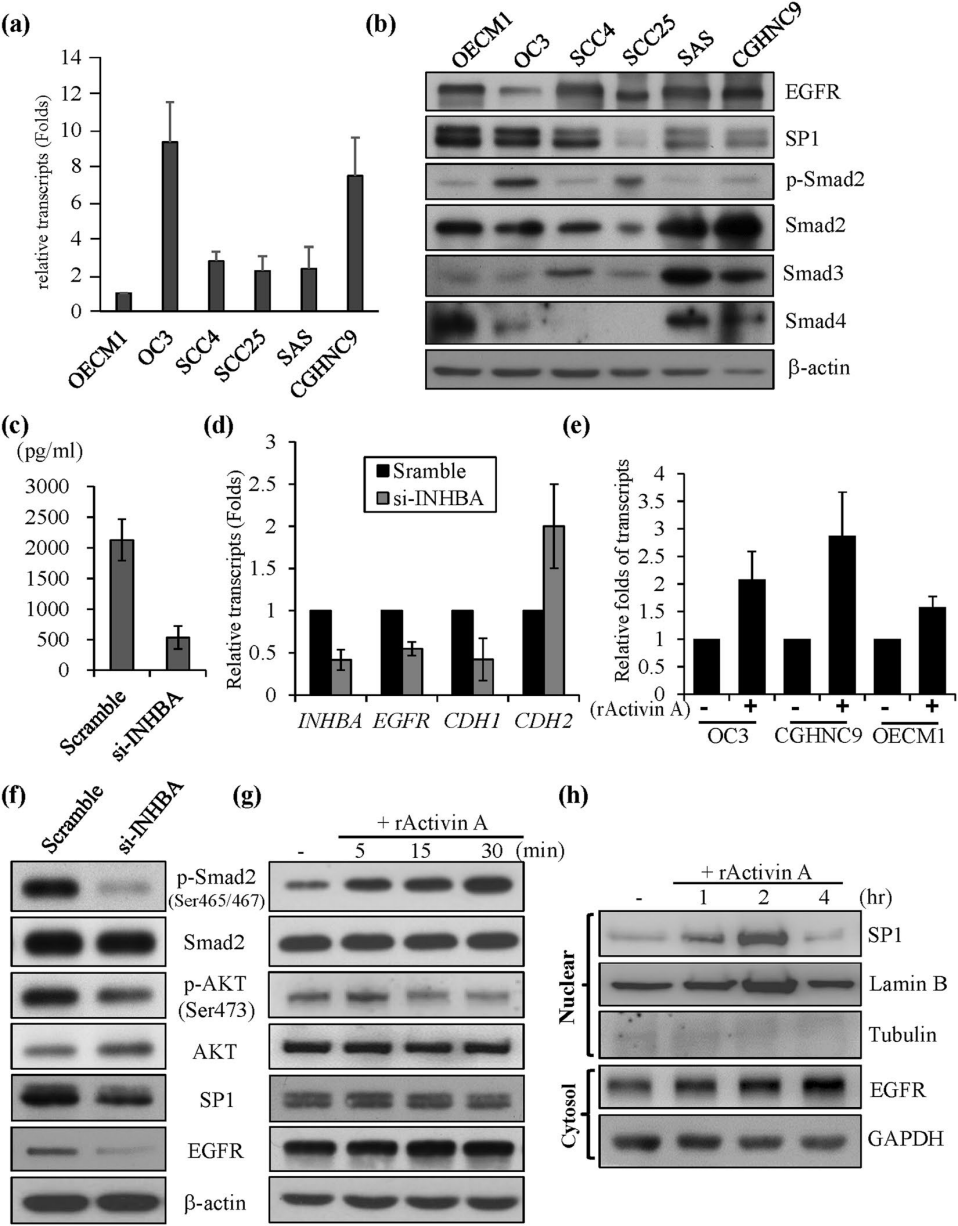
Knockdown of the endogenous expression of *INHBA* using RNAi abolished p-Smad2, attenuated p-AKT ser473, and repressed the expression of SP1 and EGFR in OSCC cell lines. (**a**) Transcripts of *INHBA* were measured by performing qRT-PCR using a specific primer/probe set and normalized to the expression level of *GAPDH*. Transcripts of *INHBA* in OECM1 cells were considered onefold (1X), and the relative folds of the *INHBA* transcripts were estimated in comparison to the endogenous *INHBA* level in OECM1 cells. (**b**) Protein expression of EGFR, SP1, p-Smad2, Smad2, Smad3, and Smad4 was analyzed in six OSCC cell lines via western blotting. (**c**) Secreted activin A protein levels in the supernatants of scramble- or si-*INHBA*-transfected OC3 cells were measured using a specific ELISA kit (R&D). (**d**) Transcripts of *INHBA*, *EGFR*, *SP1*, *CDH1* and *CDH2* were determined by performing qRT-PCR using specific primers/probes and normalized to the expression level of *GAPDH* either in the scramble- or si-*INHBA*-transfected OC3 cells. The expression level of each gene was considered onefold in scramble control cells, and the relative expression fold of each gene was normalized to that of the scramble control cells. (**e**) Transcripts of *EGFR* in OC3, OECM1, and SAS cells were determined by performing qRT-PCR using specific primers/probes, and the data were normalized to the expression of *GAPDH*. Each cell group without rActivin A treatment was considered onefold. (**f**) The expression of p-Smad2/Smad2, p-AKT (ser473)/total AKT, SP1 and EGFR in OC3 cells transfected with Scramble/si-INHBA was examined by western blotting. (**g**) OC3 cells were exogenously treated with/without rActivin A for various times, followed by western blot analysis to detect p-Smad2/Smad2, p-AKT/AKT, SP1 and EGFR. (**h**) OC3 cells were treated with rActivin A for 1, 2, and 4 hr following nuclear and cytosol fractionation. The expression of SP1, lamin B, tubulin, and EGFR in the nuclei or cytosol was examined via western blotting. GAPDH, β-actin, lamin B, and tubulin expression served as internal controls.

The endogenous levels of activin A, EGFR, the canonical Smad family and the transcription factor SP1 were evaluated by performing quantitative real-time PCR (qRT-PCR) or western blotting in OSCC cell lines (Fig. 3a,b). Among six OSCC cell lines, OC3, CGHNC9 and OECM1 cells were chosen for use in this study because they were isolated from patients with buccal cancers in Taiwan with similar etiologies, such as betel nut chewing^32,33^. The OC3 and CGHNC9 cells expressed the highest transcript levels of *INHBA*, whereas OECM1 cells expressed the lowest transcript levels of *INHBA* (Fig. 3a). At first glance, p-Smad2 expression in OC3 cells was highest among the OSCC cell lines, while EGFR expression was lowest in OC3 cells (Fig. 3b).

Endogenous *INHBA* transcripts were knocked down in OC3 cells via specific RNAi, and activin A protein secretion in the culture supernatant was reduced by 80% (Fig. 3c) following the repression of 60% of endogenous *INHBA* transcripts in si-*INHBA*-transfected OC3 cells compared to that in the scramble control cells (Fig. 3d). In si-*INHBA*-transfected OC3 cells, *CDH1* transcripts were repressed, and *CDH2* was upregulated, which is consistent with a previous report showing that activin A could induce epithelial-mesenchymal transition (Fig. 3d)^14^. The transcripts of *EGFR* were also coordinately reduced in si-*INHBA*-transfected OC3 cells compared to those in the scramble control cells (Fig. 3d). Furthermore, *EGFR* transcripts were activated in three cell lines treated with exogenous rActivin, as revealed by qRT-PCR (Fig. 3e). Therefore, activin A modulated *EGFR* via transcriptional control in OSCC cell lines.

To identify the signaling involved in *EGFR* transcriptional activation, p-Smad2(ser465/467) and p-AKT(ser473) were significantly repressed in si-*INHBA*-transfected OC3 cells compared to scramble control cells, indicating that the knockdown of endogenous *INHBA* transcripts attenuated downstream canonical Smad-dependent and noncanonical PI3K signaling (Fig. 3f). Meanwhile, the expression of SP1 and EGFR was also reduced in si-*INHBA*-transfected OC3 cells compared to that in the scramble control cells (Fig. 3f). For further confirmation, OC3 cells were treated with exogenous rActivin A at various time points, and p-Smad2 was rapidly activated after the cells were treated with rActivin A for 5–15 min (Fig. 3g), whereas the expression of SP1 and EGFR was activated after rActivin A treatment for 1–2 and 4 hr, respectively; furthermore, SP1 levels in the nucleus increased in OC3 cells (Fig. 3h). These findings were also consistent with those from CGNHC9 cells treated with exogenous rActivin A (Fig. S2). Thus, activin A-mediated activation of canonical Smad and noncanonical PI3K correlated with elevated SP1 and EGFR expression in OSCC cell lines.

### SP1 was essential for EGFR activation, and canonical Smads acted as inhibitory regulators of the EGFR proximal promoter

To analyze the role of canonical Smad, noncanonical PI3K signaling and SP1 in *EGFR* regulation, site-directed mutagenesis was conducted for SP1 (EGFR-418/+107-Luc mSP1) and the SBE responsive element (EGFR-418/+107-Luc mSBE); these two constructs were generated in addition to the insertion of the *EGFR* promoter in front of a TATA-less luciferase reporter construct (Fig. S1).

**Figure 4:**
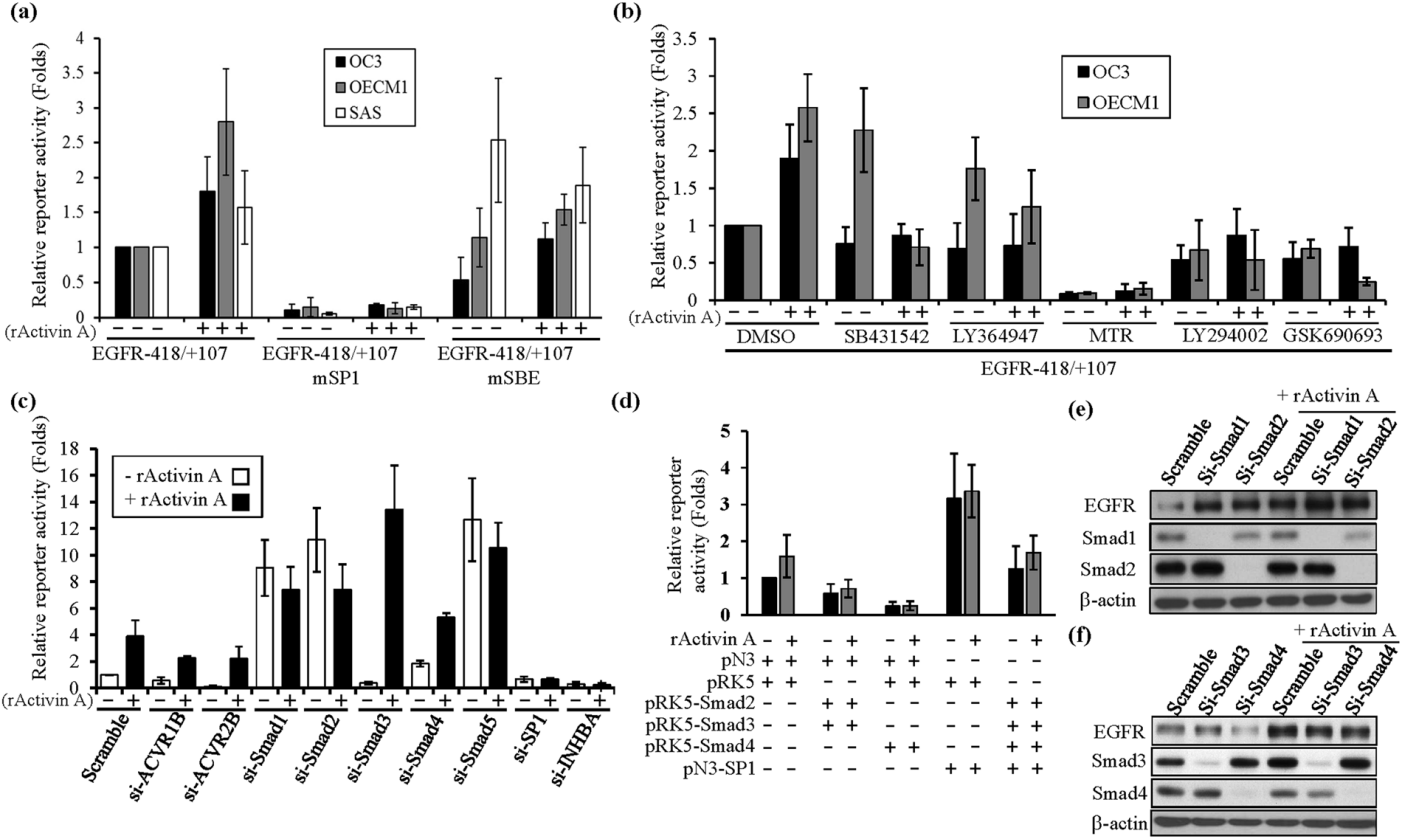
The SP1 and Smad transcription factors were crucial for rActivin-mediated regulation of the EGFR promoter as measured using a luciferase reporter assay. (**a**) Luciferase reporters were transfected into OC3, OECM1 and SAS cells with or without rActivin A treatment. All luciferase activity was normalized to Renilla reporter activity. The normalized reporter activity in cells transfected with EGFR-418/+107 without rActivin treatment (control) was considered onefold, and the relative fold for each construct (mSP1 or mSBE) with or without rActivin A treatment was further normalized to that of the control. (**b**) Reporters were transfected into OECM1 and OC3 cells with or without rActivin versus treatment with SB431542, Mithramycin, LY294002 and GSK690693. The normalized reporter activity in cells transfected with EGFR-418/+107 without rActivin treatment (DMSO, vehicle) was considered onefold, and the relative fold of each treatment was calculated. (**c**) The EGFR-408/+107 luciferase reporter was cotransfected with scramble, si-*INHBA*, si-*SP1*, si-*ALK4*, si-*ACVR2B*, *si-Smad1*, si-*Smad2*, si-*Smad3*, si-*Smad4 or si-Smad5* in OECM1 cells treated with or without rActivin A. The normalized reporter activity in cells transfected with EGFR-418/+107 without rActivin treatment was considered onefold, and the relative fold of each transfection was calculated. (**d**) The EGFR promoter (EGFR-408/+107) was cotransfected with vectors (pN3 and pRK5), pRK5-Smads, or pN3-SP1 in the presence or absence of rActivin A in OC3 cells. The normalized reporter activity in cells transfected with EGFR-418/+107 and vectors (pN3 and pKR5) without rActivin treatment was considered onefold, and the relative fold of each transfection was calculated. (**e**,**f**) Protein expression of EGFR, Smad1, Smad2, Smad3, Smad4 and SP1 was examined in scramble- and siRNA-transfected OC3 cells via western blotting. Expression of β-actin was used as an internal control. The complete medium was changed to serum-free conditions 24 hr after transfection, and cells were treated with rActivin A for 24 hr before harvesting. Each luciferase experiment is presented as the average of three to five individual experiments.

The *EGFR* proximal promoter (EGFR-418/+107) and mutants were transfected into OC3, OECM1 and SAS cells treated with or without rActivin A, and promoter activity increased by 1.5- to 2-fold in the presence of activin A compared to that without treatment (Fig. 4a). Thus, the *EGFR* promoter is activated via activin A in OSCC cell lines. This activation was abolished when the SP1 responsive element on the *EGFR* proximal promoter was mutated; however, the mutation of SBE alone reduced *EGFR* promoter activity to only 40% of that observed for the parental constructs in OC3 cells. It was slightly elevated *EGFR* promoter activity in OECM1 and SAS cells and showed a reduced response to rActivin A treatment in EGFR-418/+107mSBE reporter (Fig. 4a). For further confirmation, the EGFR-408/+107 luciferase reporter was transfected into OC3 and OECM1 cells treated with vehicle, SB431542 (TGF-β superfamily type I and ALK4/5/7 inhibitor), LY364947 (TGF-β type I receptor inhibitor), mithramycin A (MTR) (SP1 inhibitor), GSK690693 (pan-AKT inhibitor) and LY294002 (PI3K inhibitor) with or without rActivin A stimulation (Fig. 4b). *EGFR* proximal promoter activity was repressed by 50 ~ 80% in the MTR-, GSK690693- or LY294002-treated cells, even in the presence of rActivin A, compared to in the DMSO-treated control cells; nevertheless, SB431542 and LY364947 treatment alone elevated EGFR activity, but rActivin A-mediated *EGFR* activation was attenuated (Fig. 4b). These data implied that rActivin A-mediated *EGFR* promoter activation was ALK4 dependent, although noncanonical PI3K signaling and SP1 were also required for its activation; nevertheless, the role of canonical Smads remains to be elucidated.

To further examine the role of the Smads in *EGFR* promoter modulation in OSCC cells, the EGFR-408/+107 luciferase reporter was cotransfected with different specific siRNAs into OECM1 cells to perform a luciferase assay (Fig. 4c). The transfection efficiency of each siRNA in the OSCC cells was examined via western blotting (Fig. 4e,f). Knockdown of endogenous *INHBA*, *SP1*, *Activin A Receptor Type 2B (ACVR2B)*, and *Activin A receptor type 1B* (*ACVR1B; ALK4*) attenuated rActivin A-mediated activation of *EGFR* promoter activity (Fig. 4c). The repression of endogenous *Smad1, Smad2, Smad3, Samd4* and *Smad5* elevated *EGFR* promoter activity in the presence of rActivin A compared to that observed with scramble control cells, suggesting that ACVR2B/ALK4-mediated canonical Smads play an inhibitory role in *EGFR* promoter regulation (Fig. 4c). Consistent with the luciferase data, knockdown of endogenous Smads elevated EGFR expression, particularly following the rActivin A treatment in OSCC cells, as revealed via western blotting (Fig. 4e,f). The knockdown of endogenous SP1 expression attenuated activin A-mediated EGFR activation (Fig. 4c), suggesting that SP1 is essential for EGFR activation following activin A stimulation. The expression of EGFR was further confirmed to be modulated via overexpression of an SP1-expressing plasmid; inhibition of SP1 via its RNAi or inhibitor in OSCC cells repressed *EGFR* and blocked rActivin activation (Fig. S3).

We further transfected flag-tagged Smads and evaluated their role in *EGFR* regulation. As shown in Fig. 4d,the overexpression of flag-tagged Smad2, Smad2/3, and Smad4 alone repressed *EGFR* promoter activity and responsiveness to rActivin A, and this repression was restored by overexpressing the transcription factor SP1.

Taken together, activin A-mediated *EGFR* activation was ACVR2B/ALK4 dependent, noncanonical PI3K signaling and SP1 were essential for its activation, and mutation of the SP1 binding site or treatment with a PI3K, pan-AKT or SP1 inhibitor abolished this activation, whereas the canonical Smads acted as inhibitory regulators of the *EGFR* proximal promoter during activin A stimulation.

### Inhibition of PI3K and SP1 using specific inhibitors blocked activin A-mediated *EGFR* activation and attenuated cell migration

**Figure 5:**
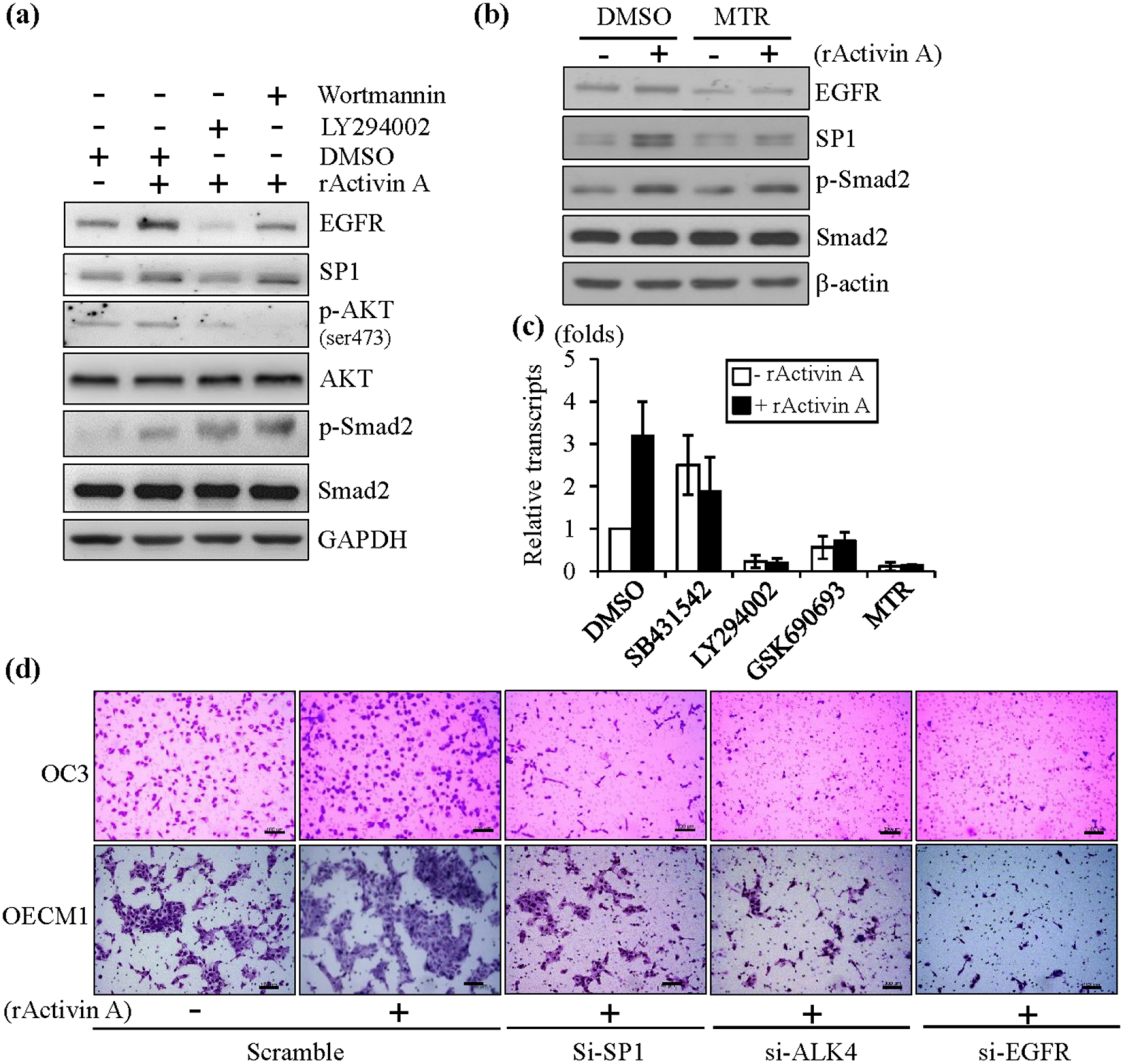
Blocking SP1 expression attenuated the expression of EGFR and repressed the migration ability of OSCC cells. (**a**) Protein expression of EGFR, SP1, p-Smad2, Smad2, SP1, p-AKT (ser473), and AKT was examined in DMSO-treated OC3 cells with or without rActivin A or Wortmannin-, LY294002-treated OC3 cells with rActivin A. GAPDH expression was used as an internal control for western blotting. (**b**) Protein expression of EGFR, p-Smad2, Smad2, and SP1 was examined in DMSO-treated OC3 cells with or without rActivin A or MTR-treated OC3 cells with rActivin A. β-actin expression was used as an internal control for western blotting. (**c**) EGFR transcripts were examined in DMSO-treated OC3 cells with or without rActivin A in the presence of SB431542, LY294002, GSK690693 or MTR. The expression of β-actin was used as an internal control in qRT-PCR. (**d**) Inhibition of *SP1*, *ALK4* and *EGFR* expression via RNAi repressed the rActivin A-induced cell migration ability of OSCC cells, as revealed via a transwell migration assay (scale bar: 100 µm).

To further exam the effects of PI3K signaling and SP1 on EGFR activation via activin A, the specific inhibitors LY294002, and Wortmannin were applied to OSCC cells treated with rActivin A. As shown in Fig. 5a, the endogenous expression of p-AKT (ser473), SP1 and EGFR was repressed in OC3 cells treated with LY294002, and Wortmannin but expression of p-Smad2, total Smad2 and total AKT remained unchanged after these treatments (Fig. 5a). Meanwhile, treatment with MTR blocked SP1 binding and reduced its expression in OC3 cells and impaired EGFR activation in the presence of rActivin A (Fig. 5b). To further confirm that PI3K/SP1 signaling affected EGFR transcripts, EGFR expression was measured via qRT-PCR with or without rActivin treatment in the presence of ALK4, PI3K, AKT and SP1 inhibitor in OC3 cells. As shown in Fig. 5c, EGFR transcripts were activated in the presence of rActivin A, and this activation was attenuated in the presence of ALK4 inhibitor and suppressed by the PI3K, pan-AKT and SP1 inhibitors (Fig. 5c). Therefore, these data indicate that activin A activated EGFR through ACVR2B/ALK4/PI3K/SP1 signaling in OSCC cells.

Finally, to demonstrate that activin A-mediated ACVR2B/ALK4/PI3K/SP1 EGFR activation is essential for cell migration, endogenous ALK4, SP1 and EGFR were knocked down using specific RNAi in OSCC cells with or without rActivin A treatment. As shown in Fig. 5d, the cell migration abilities of OSCC cells were enhanced through exogenous rActivin A stimulation, and this rActivin A-induced cell migration was attenuated following the repression of ALK4, SP1 and EGFR expression in OSCC cells. Altogether, the *EGFR* promoter was regulated by activin A through PI3K/SP1 signaling, whereas the ALK4-mediated canonical Smad pathway was a negative regulator of the *EGFR* promoter. Activin A-mediated activation of ACVR2B/ALK4/PI3K/SP1 was crucial for EGFR activation, which in turn increased cell migration in OSCC cells.

### Recruitment of SP1, CBP, P300 and Smads to the EGFR promoter in the presence of activin A

**Figure 6:**
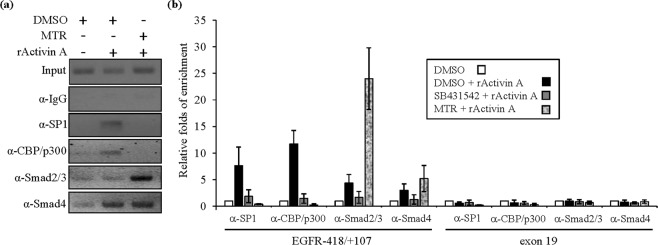
A transcriptional complex consisting of Smad2, Smad4, SP1, and CBP/p300 was recruited to the EGFR promoter in the presence of rActivin A as demonstrated by ChIP followed by quantitative real-time PCR. (**a**) The gel electrophoresis results for a ChIP assay followed by quantitative real-time PCR. ChIP DNA fragments bound by various antibodies were amplified by a specific primer targeting the EGFR proximal promoter (−418/+107), as measured by a ChIP assay followed by qRT-PCR, for which products were separated on a 2% agarose gel. (**b**) The results are expressed as the relative fold of enhancement for quantitative real-time PCR following the ChIP assay. OC3 were cells treated with vehicle (DMSO) with or without rActivin A or with inhibitors in the presence of rActivin A. Formaldehyde-fixed DNA was pulled down by various antibodies, followed by quantitative real-time PCR analysis. The EGFR exon 19 PCR served as a nonspecific control for the ChIP assay; an IgG control was also used as a negative control for the ChIP assay, while ChIP DNA fragment pull-down using α-acetyl-Histone 3 was a positive control for the active promoter in this assay (not shown in this figure). Calculations were performed as described in the Materials and Methods.

To demonstrate that the transcription factor SP1 and the Smads bind the *EGFR* proximal promoter, OC3 cells were treated with specific inhibitors in the presence or absence of rActivin A, followed by a chromatin immunoprecipitation (ChIP) assay and real-time PCR. The transcription factor SP1, Smad2/3, Smad4, and the coactivator CBP/p300 were detected via ChIP following real-time PCR using primers targeting the *EGFR* proximal promoter (−418/+107) (Fig. 6a) and its exon 19 region (as a nonspecific control)^27^. Meanwhile, the transcription factor SP1, Smad2/3, Smad4, and CBP/p300 increased recruitment to the *EGFR* proximal promoter by approximately four- to fivefold following rActivin A treatment in OC3 cells as compared to that in untreated cells (Fig. 6a,b). This association complex was not detected using *EGFR* exon 19 PCR primers (Fig. 6b). The abovementioned transcriptional complex was dramatically altered in OC3 cells treated with rActivin A in the presence of MTR, the binding of the transcription factor SP1 and CBP/p300 to the *EGFR* proximal promoter decreased, and the binding of Smad4 was slightly elevated up to fivefold. However, the binding of Smad2/3 to the EGFR proximal promoter dramatically increased by nearly 24- to 30-fold compared to that in untreated cells (Fig. 6b). In contrast, treatment with the ALK4 inhibitor SB431542 attenuated the recruitment of Smad2/3/4 on the *EGFR* proximal promoter and repressed the binding of SP1 and CBP/p300 to the *EGFR* promoter following rActivin A treatment (Fig. 6a,b). Thus, the SP1, CBP/p300 and Smad2/3/4 complex was recruited to the *EGFR* proximal promoter following rActivin A treatment. Prevention of the binding of SP1 and CBP/p300 to the consensus sequences of the *EGFR* proximal promoter via treatment with an inhibitor targeting SP1 increased the binding of Smad2/3/4 to the SBE adjacent to the SP1 binding site, suggesting canonical Smads acted as negative regulators by outcompeting the SP1 consensus in the *EGFR* promoter.

In conclusion, activin A activates the transcription factor SP1 and Smads through canonical and noncanonical pathways, respectively; both modulate EGFR promoter activity but have opposite effects in OSCC cells.

## Discussion

EGFR is an important therapeutic target in various cancers, including OSCC, and a mutation in its kinase domain is a primary cause of relapse^34^. The expression of EGFR is modulated by transcriptional, posttranscriptional, posttranslational control and ligand stimulation^22,35–38^. Mutations in EGFR enhance the activation of its downstream signaling, and some mutations also facilitate drug escape through endocytosis and increasing protein stability^35–37^. However, the rate of *EGFR* mutations in OSCC, which ranges from 2.7% to 8%, is lower than that in other cancers (S1 Table)^39–41^. Most of the mutations in *EGFR* are not functionally validated in oral cancers, and the well-known mutations associated with tyrosine kinase inhibitor (TKI) sensitivity/resistance, such as G719X, T790M, exon 19 deletion/insertion, exon 20 insertions, L858R and L861Q, have not been found in OSCC^34,41^. Therefore, the role of mutations in EGFR stability remains to be experimentally elucidated, although its impact might be minor due to the low frequency of mutations in OSCCs (Table S1)^39–41^.

In addition to EGFR, the rate of Smads mutations is also low in OSCC tissues, although current evidence suggests that the deletion of Smad4 contributes to the metastasis of head and neck squamous cell carcinoma (HNSCC). Smad4 mutations only account for 1–4% of HNSCC cases (Table S1)^39–41^. p-Smad2 expression was lower in tumors than in their normal tissue counterparts (Fig. S4), suggesting that these canonical Smads are largely repressed in OSCC tissues, which is consistent with a previous report that highlighted the absence of activated Smad2 in HNSCC^42^. Therefore, the activin A-mediated noncanonical pathway should be crucial for activation of the EGFR promoter since negative regulator canonical Smads were decreased in tumor tissues. The growth inhibition effects of these canonical Smads might be diminished during carcinogenesis due to mutation or elevation of inhibitory Smads (Smad6, Smad7)^43,44^; however, we did not evaluate the role of inhibitory Smads in our study, although these inhibitory Smads are overexpressed in HNSCC^44^. Therefore, the interaction between inhibitory Smads, PI3K/AKT activation, SP1 expression and EGFR activation should be further examined in OSCC cells in the future. Nevertheless, the absence of canonical pathways in cancer cells prevents growth inhibitory effects derived from canonical Smads^15,18,45^, and the noncanonical pathways derived from activin A should be critical for OSCC carcinogenesis.

In our study, the activin A-mediated noncanonical pathway-PI3K/SP1 was essential for the carcinogenesis of OSCC. Among six OSCC cell lines, the Smad4 deletion was found in SCC4 and SCC25 cells but not in the cell lines derived from Taiwanese patients with areca chewing etiology (OC3, OECM1 and CGHNC9 cells) (Fig. 3b), indicating that different etiologies or ethnic factors likely play roles in the carcinogenesis of OSCC. Indeed, areca chewing is strongly associated with pathological alterations in the oral mucosa, including oral submucous fibrosis (OSF), leukoplakia, erythroplakia, and even cancers^46^. The detailed carcinogenesis mechanism arising from the areca nut (AN) indicates AN is a group I human carcinogen^47^. Additionally, repeated mucosal damage during chewing mechanistic pressure results in wound-healing processes and the upregulation of inflammatory cytokines^48^. Among these cytokines, TGF-β, activin A and activin B are overexpressed in either cancer tissues or cancer-associated fibroblast cells in OSCCs or HNSCC^16,49–51^. Indeed, the Activin family has been demonstrated to promote skin wound healing *in vivo*^52–54^. This information seems to highlight that AN chewing or AN extract itself might be linked to activation of the ACVR2B/ALK4/PI3K/SP1/EGFR pathway; however, this possibility should be further validated using animal models in the future.

Several pro-inflammatory cytokines, including TGF-β and activin A, can trigger the PI3K/AKT/SP1 pathway, which further activates various downstream target genes through SP1 binding to GC-rich consensus sequences, including *cystathionine-γ-lyase* (*CSE*), *purinergic P2X7 receptor* (*P2X7R*), *VEGF*, *insulin-like growth factor binding protein 2* (*IGFBP-2*), *E-prostanoid receptor 4* (*EP4*) and *EGFR*^55–60^. Thus, several ongoing clinical trials are attempting to block PI3K/AKT/SP1 pathway activation using specific inhibitors to attenuate cancer cell proliferation, cell migration, and tumor invasion. A recent report meta-analyzed 46 studies and showed that targeted therapy against PI3K/AKT signaling as an adjuvant treatment for advanced solid tumors significantly improves progression-free survival^61^. Additionally, the signaling inhibitor MTR has been tested for cancer therapy since the 1960s due to its ability to prevent SP1 and SP3 binding but has never been accepted for cancer treatment due to its broad-spectrum effects in blocking RNA and protein synthesis^62^, resulting in side effects including hepatoxicity, gastrointestinal symptoms, and renal toxicity^62^. Recently, some reports showed that improved MTR analogs could significantly reduce side effects in patients and demonstrate promising anti-proliferation and sensitizing effects as adjuvant treatments for several kinds of cancer cells, including esopharyngeal cancers^63–68^. Currently, we are trying to establish a PDX mouse model from samples from OSCC patients from the southeastern area to study OSCC tumors in patients and drug responses.

Activin A is significantly associated with EGFR in OSCC tissues at both the transcript and protein levels. The high expression of EGFR is also associated with many poor prognosis factors and lower posttreatment survival in OSCC patients. The *EGFR* promoter is activated by activin A in OSCC cells via a noncanonical pathway in which ACVR2B/ALK4/PI3K/SP1, SP1 and CBP/p300 are recruited to the promoter. The canonical Smad pathway serves as a negative regulator of *EGFR* expression and is largely suppressed in OSCC tumors, suggesting the crucial role of the noncanonical pathway in OSCC carcinogenesis.

## Materials and Methods

### Patient characteristics and clinical specimens

This study was approved by the Medical Ethics Committee of Chang Gung Memorial Hospital in Linkou, Taiwan (#104-9444B). Informed consent was obtained from all participants in this study. Patients in the study underwent standard preoperative work-ups according to institutional guidelines and regulations. Tumor specimens for the immunohistochemical study were obtained from 155 consecutively enrolled patients (140 men and 15 women) with OSCC diagnosed at Chang Gung Memorial Hospital, Linkou Branch from 2005–2011. Patients with at least one of the following conditions were considered ineligible for the study: unresectable cancer, synchronous primary cancer, recurrent cancer, distant metastasis, prior history of malignancy, and treatment with neoadjuvant therapy. Patient age at diagnosis ranged from 22.0 to 83.6 years (mean, 51.8 ± 11.9). The associated subsites were buccal mucosa (59 patients), gum (24), hard palate (4), lip (5), floor of the mouth (5), and tongue (58). Patients in the study underwent standard preoperative work-ups according to institutional guidelines. After surgical treatment, the pathological TNM classification of all tumors was established according to the American Joint Committee on Cancer Staging Manual (2010). After discharge, all patients had regular follow-up visits every 2 months for the first year, every 3 months for the second year, and every 6 months thereafter.

### Luciferase assay

The EGFR proximal promoter was amplified via EGFR Promoter F (5′-CCACCGGTACCGGCGGCCGCTGGCCTTG-3′) and EGFR Promoter R (5′-CGGCGAGACACGCCCTTACCTTT-3′) using genomic DNA extracted from OC3 cells. PCR products were cloned into pCRII-TOPO vector (Thermo Fisher Scientific) and then subcloning into pGL3-basic (Promega, Wisconsin, United States), and the construct was referred to as EGFR-418/+107-Luc. Site-directed mutagenesis of SP1 and SBE was performed with the QuikChange Site- Directed Mutagenesis Kit (Stratagene) according to the manufacturer’s recommendations. The following primers were used to generate the SP1 mutation constructs on the EGFR promoter: mSP1 forward, 5′-GCCG**TT**CAGACCGGACGACA-3′, and mSP1 reverse, 5′-GTCTG**AA**CGGCGGCGGCCGC-3′. The following primers were used to generate the mSBE site: mSBE forward primer, 5′-GCCGCC**T**A**C**ACCGGACGACAGGCCACCTCGT, and mSBE reverse primer, 5′-**G**T**A**TGGCGGCGGCGGCCGC-3′; EGFR-418/+107-Luc was used as the plasmid DNA template. The OC3/OECM1 cells were seeded at a density of 3 × 10^5^ cells/per 6 wells and transfected per the manufacturer’s recommendations (Lipofectamine 3000, Invitrogen). The Renilla reporter (pRL-TK Vector, Promega) was mixed with luciferase reporters at a ratio of 1:20. The growth medium was removed from cultured cells 24 hr posttransfection, and serum-free medium was added for another 24 hr. Serum-starved cells were treated with 10 ng/ml rActivin A for 24 hr with or without specific inhibitors before harvest. The luciferase/Renilla assay was measured per the manufacturer’s suggestion (Promega) by a Victor3^TM^ plate reader (Perkin Elmer). The inhibitors mithramycin (MTR), SB431542, LY364947, GSK690693, Wortmannin and LY294002 were purchased from Sigma-Aldrich and used in OSCC cells at final concentrations of 200 nM, 10 μM, 50 nM, 10 nM, 200 nM and 10 μM, respectively.

### ChIP

The ChIP procedure was carried out according to the manufacturer’s suggestion [Chromatin Immunoprecipitation (ChIP) Assay Kit; Millipore, Darmstadt, Germany]. The pull-down DNA was amplified for detection with either EGFR forward and reverse primers (primer sequences in the luciferase assay section) or exon 19 primers^27^ by real-time PCR (Stratagene Mx3005P). The PCR products were finally separated on a 2% agarose gel with EZ-Vision DNA Dye (AMRESCO, USA). ChIP real-time PCR was quantified via the fold enrichment method (antibody signal over IgG secondary antibody control) to obtain ΔCt (Ct of IP Ab- Ct of IgG Ab). ΔΔCt was calculated as ΔCt (treatment; rActivin A or inhibitor treatment) – ΔCt (control or DMSO), and then the relative fold of enrichment was calculated as 2−^ΔΔCt^. OC3 cells were serum-starved for 24 hr before rActivin A (10 ng/ml) treatment. OC3 cells treated with DMSO alone were considered onefold, and the relative fold for each treatment was normalized to the level of the DMSO-treated OC3 group.

### Cell lines

The OC3 and OECM1 cells lines were obtained from Drs. Shu-Chun Lin and Kuo-Wei Chang (Yang Ming University, Taipei, Taiwan), respectively, and were maintained as previously described^32^. The SCC4 and SCC25 cell lines were obtained from the Bioresource Collection and Research Center (BCRC) in Hsinchu, Taiwan, and were maintained according to BCRC recommendations. The SAS and CGNHC9 cell lines were kindly provided by Dr. Ann-Joy Cheng (Chang-Gung University, Taoyuan, Taiwan)^33^.

### RNAi, shRNA and plasmid DNA transfection

The RNAi constructs used in this study are listed in S2 Table. RNAi (RNAiMax, Invitrogen, Carlsbad, CA) and plasmid transfection (Lipofectamine 3000, Invitrogen, Carlsbad, CA) were performed per the manufacturer’s recommendations. pKR5 series Smads were purchased from Addgene (http://www.addgene.org). In this study, the cells were seeded at a density of 3 × 10^5^ cells per well in six-well plates before transfection, and all transfected cells were serum-starved for 24 hr after transfection, followed by rActivin A treatment in serum-free medium, and harvested for reporter analysis, gene expression, and protein expression analyses.

### RNA extraction and qRT-PCR detection of EGFR and activin A

Fifty-five paired OSCC tumor and pericancerous normal tissues were homogenized in liquid nitrogen using a mortar and pestle and incubated with RNAzol B reagent (Tel-Test, Friendwood, TX), and total RNA was extracted according to the manufacturer’s protocol. First-strand cDNA was synthesized from 1 μg of total RNA and mixed with a reaction mixture consisting of commercially available primers (*EGFR*: Hs01076090_m1, *INHBA*, Hs00171410_m1, *CDH1*: Hs01023895_m1, *CDH2*: Hs00983056_m1, *SP1*: Hs00916521_m1, *GAPDH*, Hs99999905_m1; Assay-on-Demand, Applied Biosystems, Foster City, CA), RNase-free water, and TaqMan Universal PCR Master Mix. Quantitative real-time RT-PCR was performed and analyzed using a 7900 HT Sequence Detection System and SDS version 2 (Applied Biosystems, Foster City, CA), respectively. All experiments were performed in triplicate, and the mean fold change was calculated for each sample.

### Immunohistochemical staining

Immunohistochemical staining was performed as previously described^16^. The antibodies used in this study and appropriate dilutions are listed in S2 Table. Images of the stained slides were obtained using a ScanScope CT automated slide-scanning system (Aperio Technologies, Vista, CA). The expression of EGFR and activin A was scored using a combined scoring method that accounted for both the staining intensity and percentage of stained cells, as previously reported^69,70^. The resulting combined score was calculated as the sum of the percentage of stained cells multiplied by the intensity scores that were independently evaluated by our pathologist (Huang Y.). Immunohistochemical scores 0, 1–100, 101–200, and 201–300 were classified as levels 0, +, ++, and +++, respectively. Levels ++ and +++ were defined as protein overexpression.

### Western blotting

Cells were lysed with radioimmunoprecipitation assay (RIPA) lysis buffer, and 25–50 μg of extracted protein was used for western blot analysis. The nuclear and cytoplasmic extraction was performed via a NE-PER nuclear and cytoplasmic extraction kit (Thermo Fisher Scientific). The dilutions of the primary antibodies are listed in S3 Table. Proteins of interest were detected using an enhanced chemiluminescence system (Millipore) according to the manufacturer’s instructions.

### Enzyme-linked immunosorbent (ELISA) assays to determine activin A levels

Activin A levels in the supernatants of OSCC cells were determined using Quantikine ELISA kits for human activin A (R&D Systems), as previously described^16^. The medium of OSCC cells was changed to serum-free medium 24 hr before harvesting the supernatants. Each experiment was performed in duplicate.

### Cell migration assay

The cell migration assay was performed as previously described^16^. Each migration assay was performed in triplicate with 3 independent experiments.

### Statistical analysis

All statistical data are expressed as the mean ± SD. A Pearson’s correlation analysis was performed to measure the association between the relative signal intensity in the quantitative real-time RT-PCR analysis and the immunohistochemical staining scores of activin A and EGFR. Protein levels, cell proliferation, cell growth, and migration experiments were analyzed by performing unpaired Student’s t-tests. Associations among the various characteristics of patients and immunohistochemical scores were evaluated by performing Wilcoxon tests. All statistical analyses were performed using SAS software (version 9.1; SAS institute, Cary, NC). All patients underwent follow-up evaluations at our outpatient clinic until December 2015 or death. Survival analysis was plotted using the Kaplan-Meyer method, and differences were evaluated by performing a log-rank test. Multivariate regression analyses were performed to define specific risk factors for disease-specific survival. All P-values are 2-sided, and statistical significance was accepted at *P* < 0.05.

## Supplementary information

Supplemental information
