# Supplementary material for: Activin A regulates the epidermal growth factor receptor promoter by activating the PI3K/SP1 pathway in oral squamous cell carcinoma cells
Source: Sci Rep. 2019 Mar 26;9:5197. doi: 10.1038/s41598-019-41396-7 (PMC6435638; doi:10.1038/s41598-019-41396-7)

Activin A regulates the epidermal growth factor receptor promoter by activating the PI3K/SP1 pathway in oral squamous cell carcinoma cells

Chi-Neu Tsai[1, 2], Chia-Lung Tsai[3], Jui-Shan Yi[4, 5], Huang-Kai Kao[6], Yenlin Huang[7], Chun-I Wang[4], Yun-Shien Lee[3, 8], Kai-Ping Chang[4, 5]*

1. Graduate Institute of Clinical Medical Sciences, Chang-Gung University, Guishan Dist., Taoyuan City 33302, Taiwan
2. Department of Surgery, Chang-Gung Memorial Hospital, Guishan Dist., Taoyuan City 33305, Taiwan
3. Genomic Medicine Core Laboratory, Chang Gung Memorial Hospital, Guishan Dist., Taoyuan City 33305, Taiwan
4. Department of Otolaryngology-Head & Neck Surgery, Chang Gung Memorial Hospital, Guishan Dist., Taoyuan City 33305, Taiwan
5. Molecular Medicine Research Center, Chang Gung University, Guishan Dist., Taoyuan City 33302, Taiwan
6. Department of Plastic & Reconstructive Surgery, Chang Gung Memorial Hospital, Guishan Dist., Taoyuan City 33305, Taiwan

7. Department of Pathology, Chang Gung Memorial Hospital, Guishan Dist., Taoyuan City 33305, Taiwan
8. Department of Biotechnology, Ming-Chuan University, Guishan Dist., Taoyuan City 33348, Taiwan

*** Corresponding author**

Dr. Kai-Ping Chang, MD, PhD

Department of Otolaryngology-Head & Neck Surgery, Chang Gung Memorial Hospital & College of Medicine, Chang Gung University, Taoyuan, Taiwan No. 5 Fu-Hsing St. Kwei-Shan, Taoyuan, Taiwan, 33305. Tel.: +8863281200x3968; Fax: +8863979361

Email: dr.kpchang@gmail.com

Supplemental information

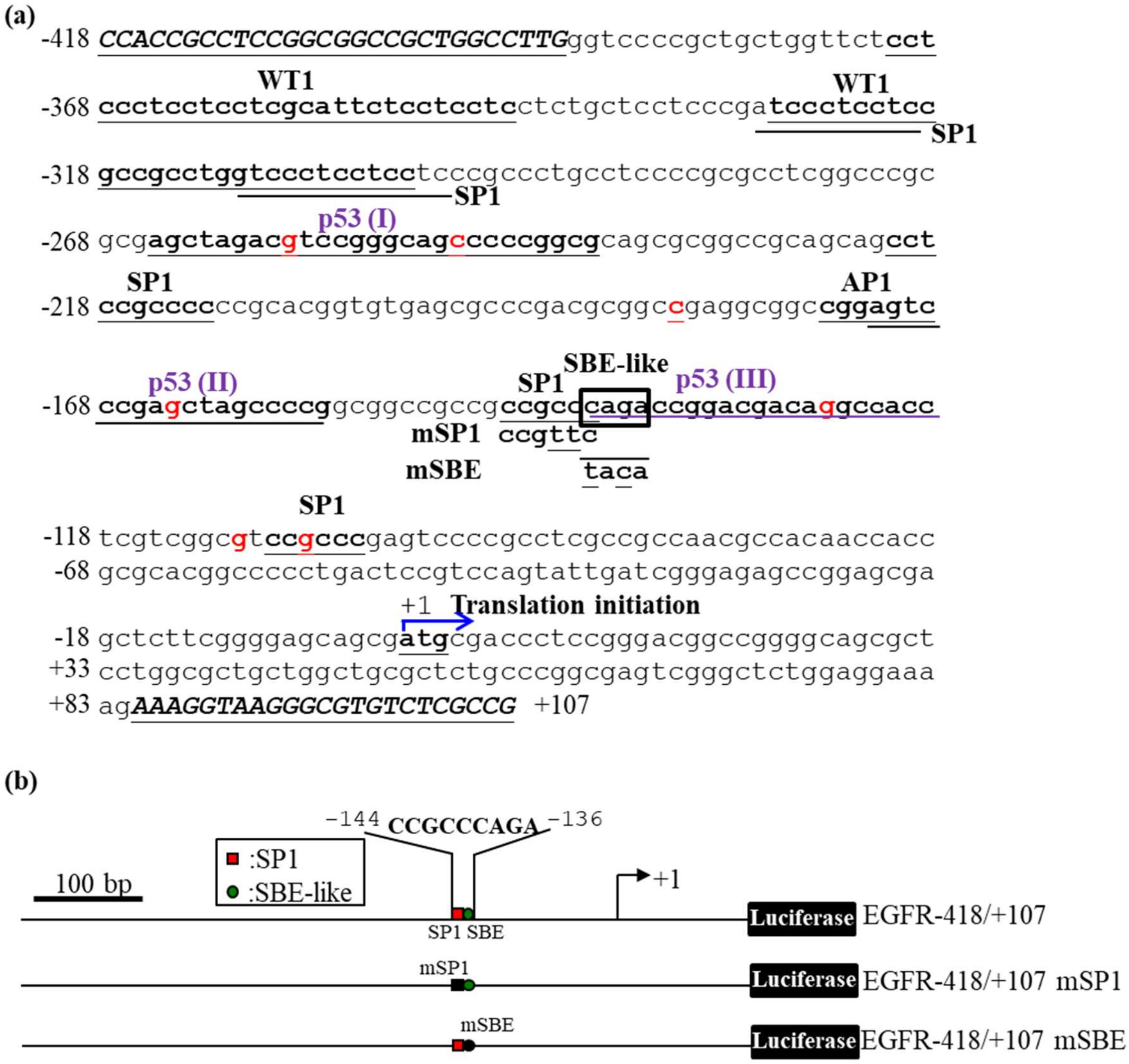

Supplemental Fig. 1. EGFR proximal promoter and luciferase constructs used in this study (a). The binding responsive element for each transcription factors, ex: SP1, AP1, WT1, and P53 were showed as underline indicated, Smad binding element–like (SBE) sequences was showed as box indicated, and the translational initiation site was labelled with +1 in the EGFR proximal promoter. Multiple transcriptional initiation sites comprise the proximal promoter of EGFR; therefore, +1 indicates the translation initiation. SP1, Smad binding consensus sequences and mutant sequences are indicated as mSP1 and mSBE. Primers used for the amplification of the EGFR promoter are shown in bold and italics. (b). Schematic representation of the EGFR proximal promoter (-418 to +107). SP1 (red colored box) and SBE-like (green colored circle) sequences are located -144 to -136 of the EGFR proximal promoter. Wild-type luciferase construct is designated EGFR-418/+107; SP1 and SBE mutant are designated mSP1 and mSBE, respectively.

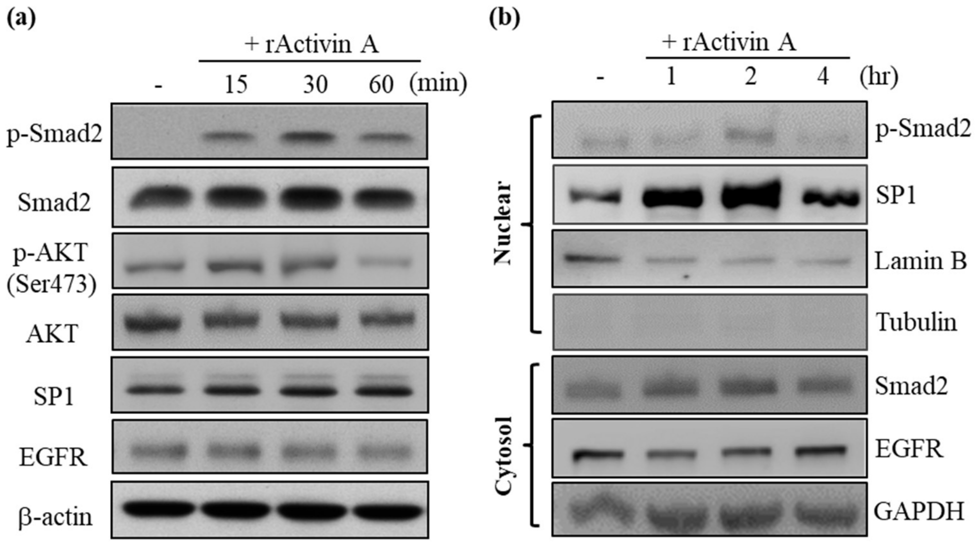

Supplemental Fig. 2. Expression of EGFR was activated following rActivin A treatment in CGHNC9 cells. (a). CGHNC9 cells were treated with/without rActivin A for various times, followed by a western blot analysis to detect p-Smad2/Smad2, p-Akt/Akt, SP1 and EGFR. Expression of β-actin was an internal control in the western blotting experiments. (b). CGHNC9 cells were treated with rActivin A for 1, 2, and 4 hours, followed by nuclear and cytosol fractionation. Expression of p-Smad2/Smad2, SP1, EGFR was examined by western blotting either in the nuclei or cytosol, expression of GAPDH and lamin B served as internal controls in the western blotting experiment. Cells were serum starved for at least 24 hours before the rActivin A treatment.

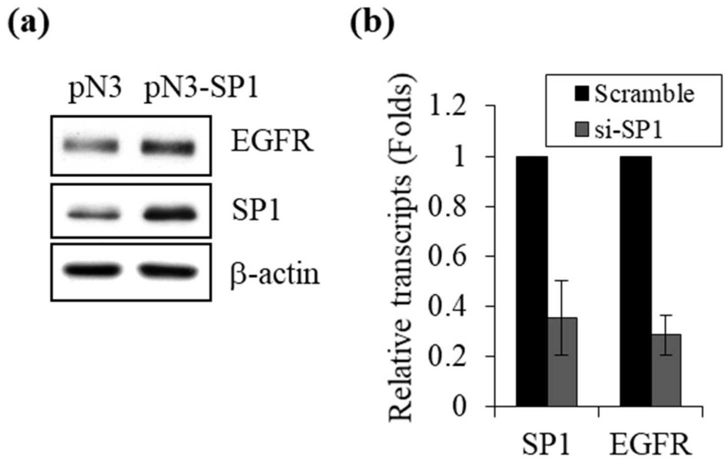

Supplemental Fig. 3. SP1 was essential for activation of EGFR transcripts/expression. (a). The SP1 expression plasmid without tagged and its vector (pN3) were transfected into OECM1, the expression of EGFR and SP1 were analyzed via western blotting. The expression of β-actin was as an internal control inwestern blotting. (b).The endogenous SP1 was knockdown via its specific RNAi in OC3 cells, the transcripts of SP1 and EGFR was measured via qRT-PCR using their specific primer/probe. The expression of SP1 and EGFR in Scramble control was considered as one fold, the relative transcripts of SP1 and EGFR in si-SP1 transfected cells were normalized with level in Scramble transfected cells.

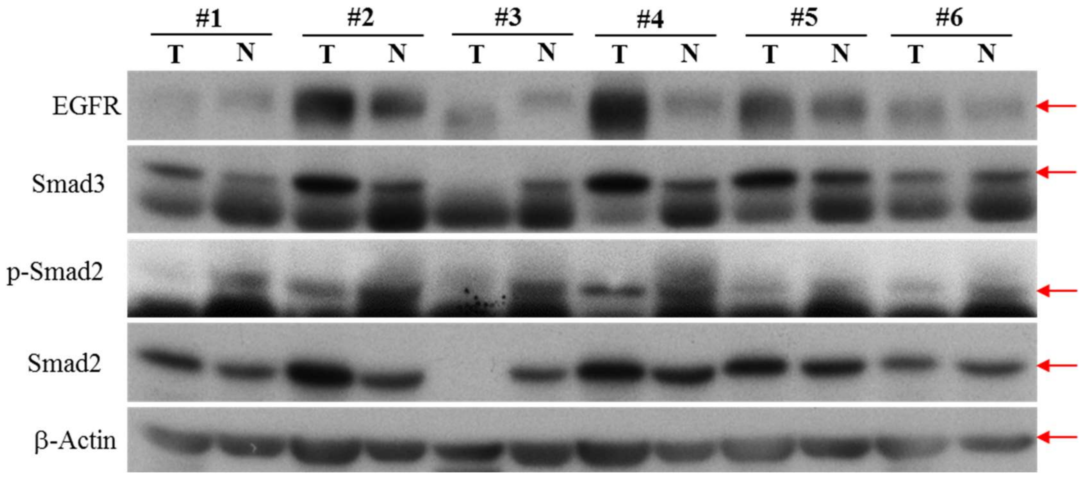

Supplemental Fig. 4. Expression of EGFR, Smad3, p-Smad2 and Smad2 was examined in six OSCC tumor (T) and adjacent normal (N) tissues. The expression of EGFR, SMAD3, p-SMAD2 and SMAD2 were examined in OSCC tumor (T) and adjacent normal (N) tissues via western blotting using their specific antibodies. Red colored arrow indicates the molecular weights of protein targets. Expression of β-actin was an internal control in the western blot analysis.

Supplemental Table 1. Frequencies of mutations in TGF-β/Activin A signaling molecules and EGFR

STUDY_NAME	Sample number	Smad1	Smad2	Smad3	Smad4	Smad5	Smad6	Smad7	ACVR1B	TGFBR1	ACVR2A	ACVR2B	EGFR
Head and Neck Squamous Cell Carcinoma (Broad, Science 2011)	74	1.40%	0%	0%	1.40%	0%	0%	0%	0%	0%	1.40%	0%	2.70%
Head and Neck Squamous Cell Carcinoma (TCGA, Nature 2015)	279	0.70%	1.10%	0.70%	2.20%	1.40%	0%	2.20%	1.40%	1.40%	1.10%	1.10%	5%
Head and Neck Squamous Cell Carcinoma (TCGA, Provisional)	510	0.60%	0.80%	0.80%	2.50%	0.40%	0.20%	2%	1%	1.40%	0.80%	1.20%	2.90%
Head and Neck Squamous Cell Carcinoma (Johns Hopkins, Science 2011)	32	0%	0%	3.10%	3.10%	0%	0%	0%	0%	0%	0%	0%	0%
Oral Squamous Cell Carcinoma (MD Anderson, Cancer Discov 2013)	40	0%	0%	2.50%	5%	0%	0%	0%	2.50%	0%	0%	0%	5%
Recurrent and Metastatic Head & Neck Cancer (JAMA Oncology, 2016)	132	0%	0.80%	0%	1.50%	0%	0%	0%	0%	3%	0%	0%	3%
Oral Cavity Squamous Cell Carcinoma (Nature Communications, 2017)	50	0%	0%	0%	2%	2%	0%	0%	2%	0%	2%	2%	8%

Supplemental Table 2. The commercial RNA interfere (RNAi) used in this study

siRNA	Company	Cat. No.
Scramble	Thermo Scientific Dharmacon	D00181010-10-20
siSmad1	Thermo Scientific Dharmacon	L-012723-00
siSmad2	Thermo Scientific Dharmacon	L-003561-00
siSmad4	Thermo Scientific Dharmacon	L003902-00-0020
siINHBA	Thermo Scientific Dharmacon	L011701-00-0020
control medium GC duplex	Invitrogen	12935-300
siSmad3	Invitrogen	HSS106252
siSmad3	Invitrogen	HSS180970
siSmad3	Invitrogen	HSS180971
siACVR1B	Invitrogen	HSS100141
siACVR1B	Invitrogen	HSS100142
siACVR1B	Invitrogen	HSS100143
siACVR2B	Invitrogen	VHS41230
siSP1	Invitrogen	HSS186048
siSmad5	Invitrogen	VHS41122

Supplemental Table 3: Antibodies used in this study and its dilution ratio

Antibody Name	Company	Cat. No.	Host	usage	western dilute
EGFR	CST	4267	r	WB	1:3000~1:5000
Beta-actin	millipore	MAB1501	m	WB	1:5000
GAPDH	Sigma	G9545	r	WB	1:5000
Smad1	CST	6944	r	WB	1:1000
SP1	CST	9389	r	WB	1:1000
Smad2	CST	5339	r	WB	1:2500
Smad2 pS465/467	CST	3108	r	WB	1:1000
Smad4	Santa cruz	sc7966	m	WB	1:200
Smad3	CST	9523	r	WB	1:1000
AKT pS473	CST	4060	r	WB	1:500
AKT pT308	CST	4056	r	WB	1:500
AKT	Santa Cruz	sc8312	r	WB	1:1000
Lamin B	Santa Cruz	sc6216	g	WB	1:2000
Tubulin	Sigma	T9026	m	WB	1:5000-1:10000
E-cadherin	millipore	07-697	r	WB	1:3000-5000
EGFR	novocastra	NCL-L-EGFR-384	m	IHC	1:100
activin A	R&D	AF338	g	IHC	1:20
Acetyl-Histone H3	millipore	06-599	r	ChIP 5ug	
p300	abcam	ab14984	m	ChIP 2ug	
SP1	Santa Cruz	sc-59x	g	ChIP 2ug	
Smad2/3	Santa Cruz	sc6033x	g	ChIP 6μg	

CST: cell signaling technology, r: rabbit, m: mouse, g: goat, WB: western, IHC: immunohistochemistry, ChIP: chromatin immunoprecipitation

All western blotting raw data

Fig3b

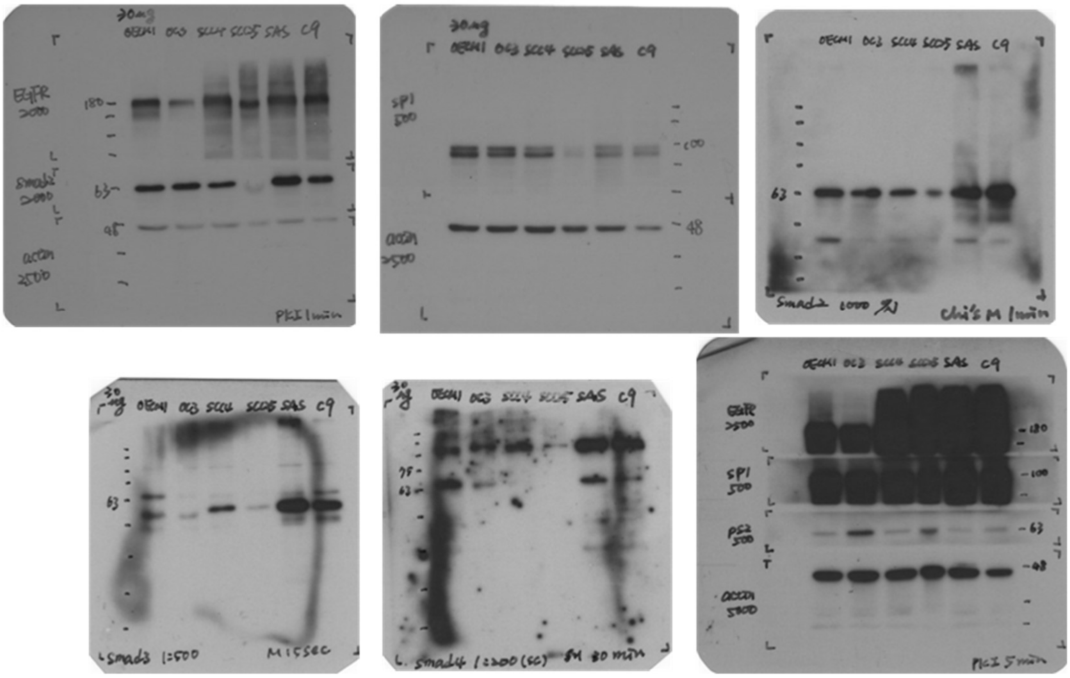

Fig3f

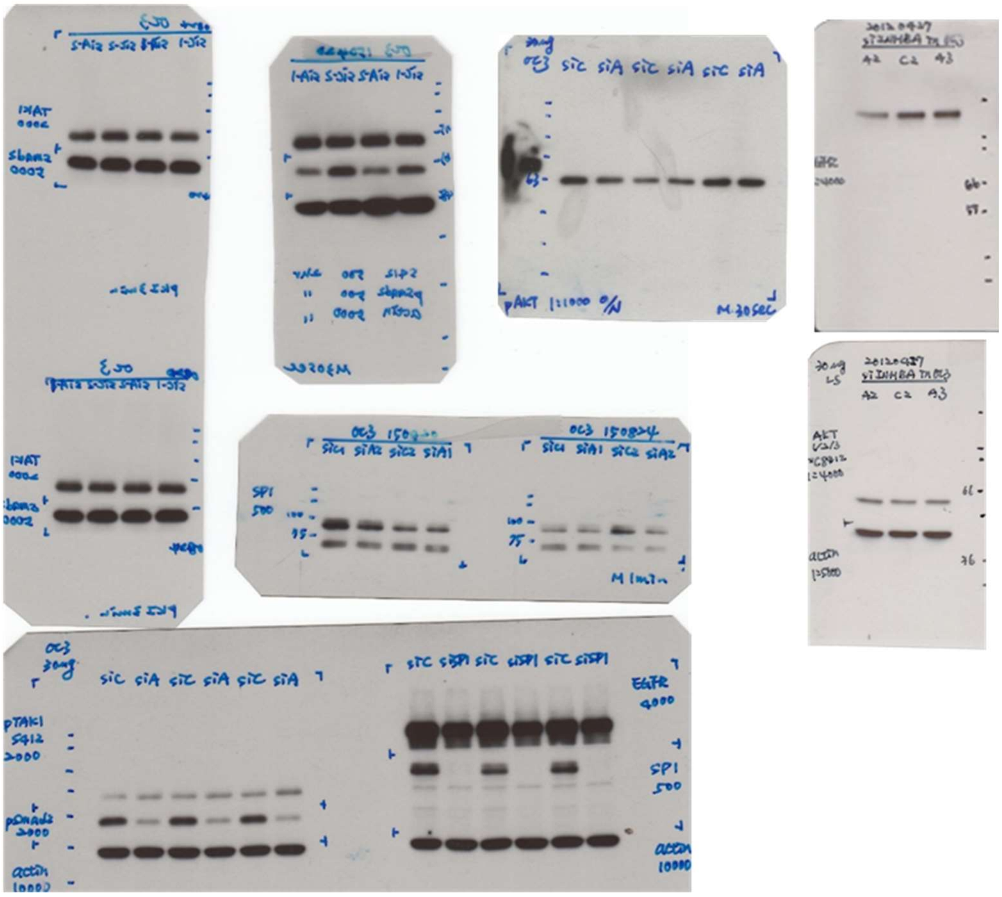

Fig.3(g)

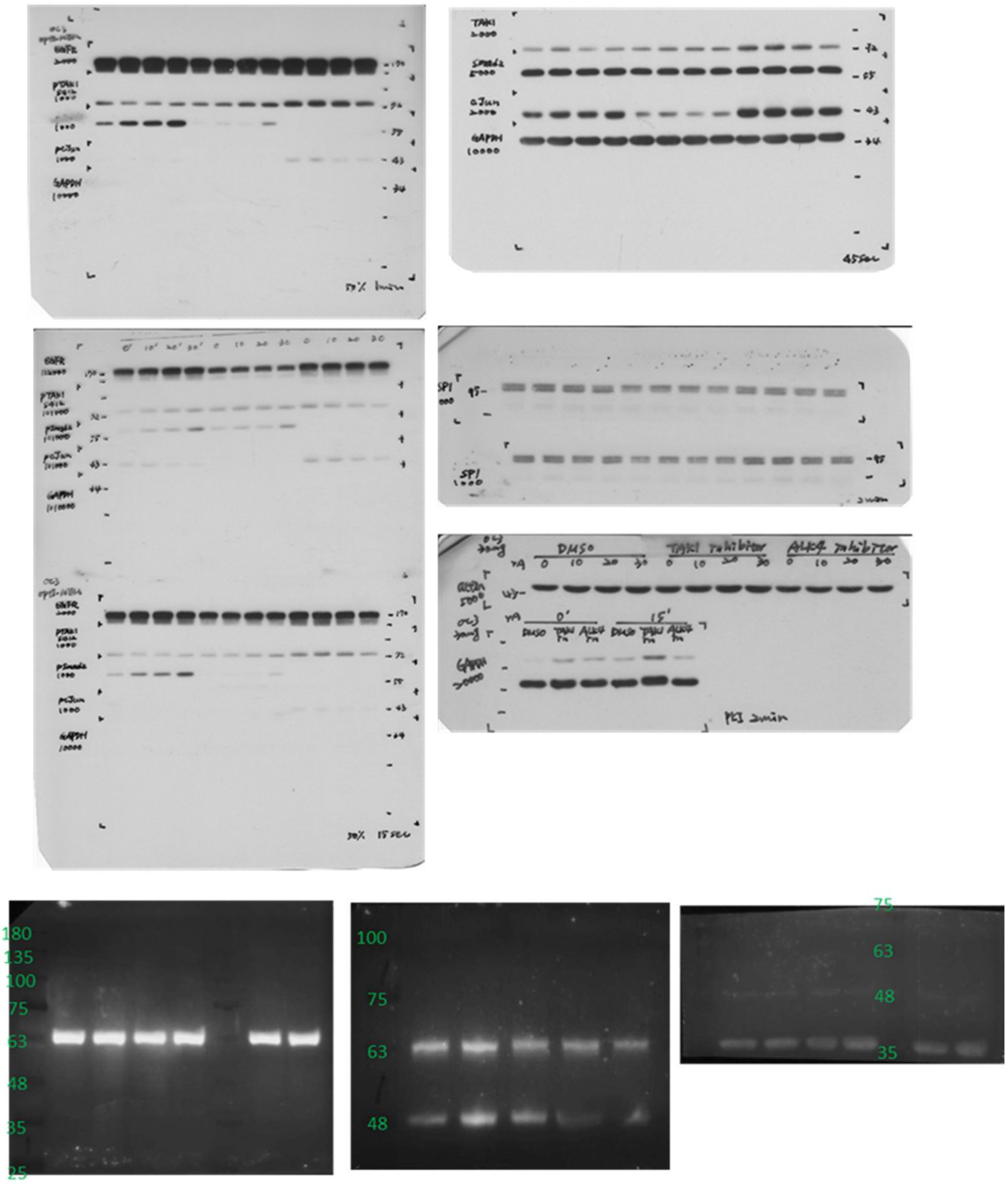
DMSO
TAK1 inhibitor
ALK4 inhibitor
180
135
100
75
63
48
35
25
100
75
63
48
75
63
48
35

Fig3h

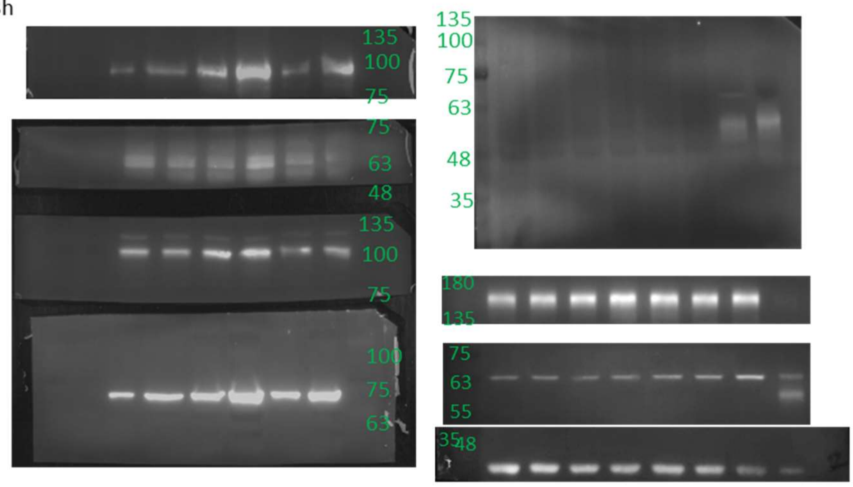

Fig4e

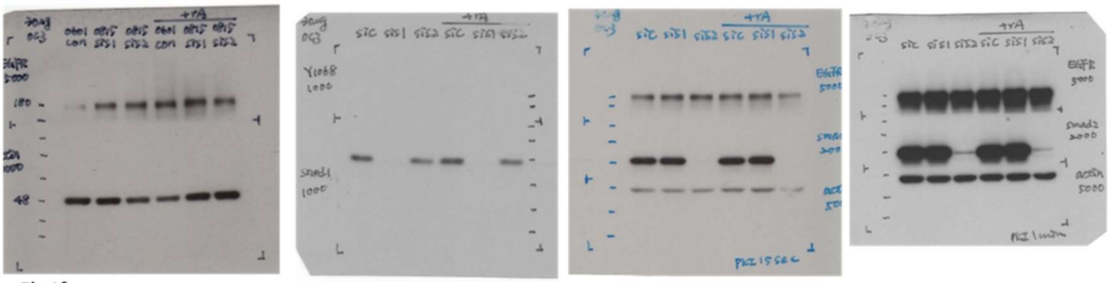

Fig4f

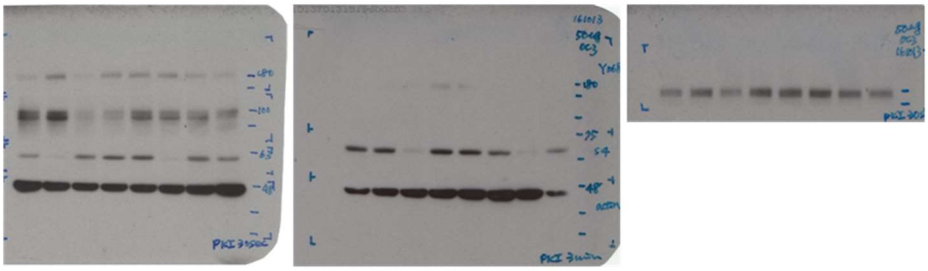

Fig. 5a

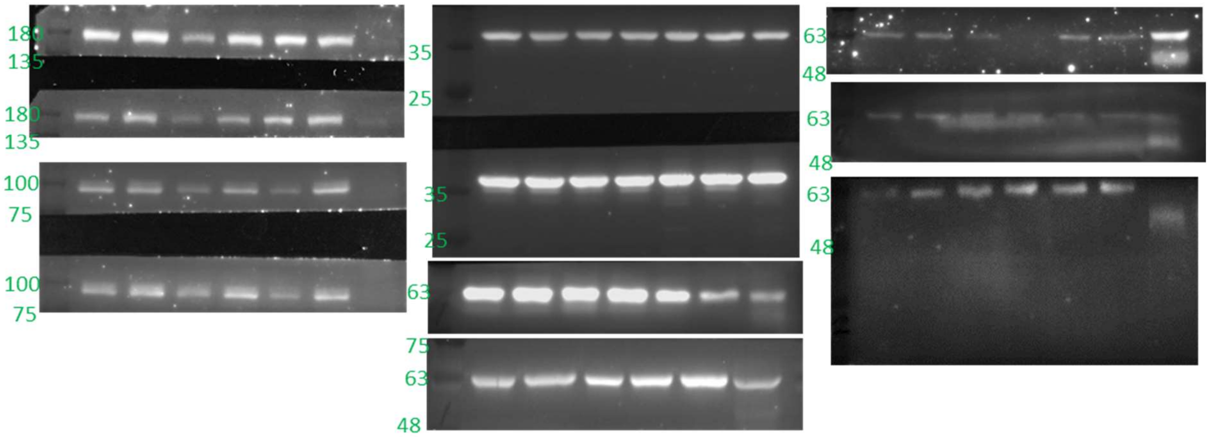

Fig. 5b

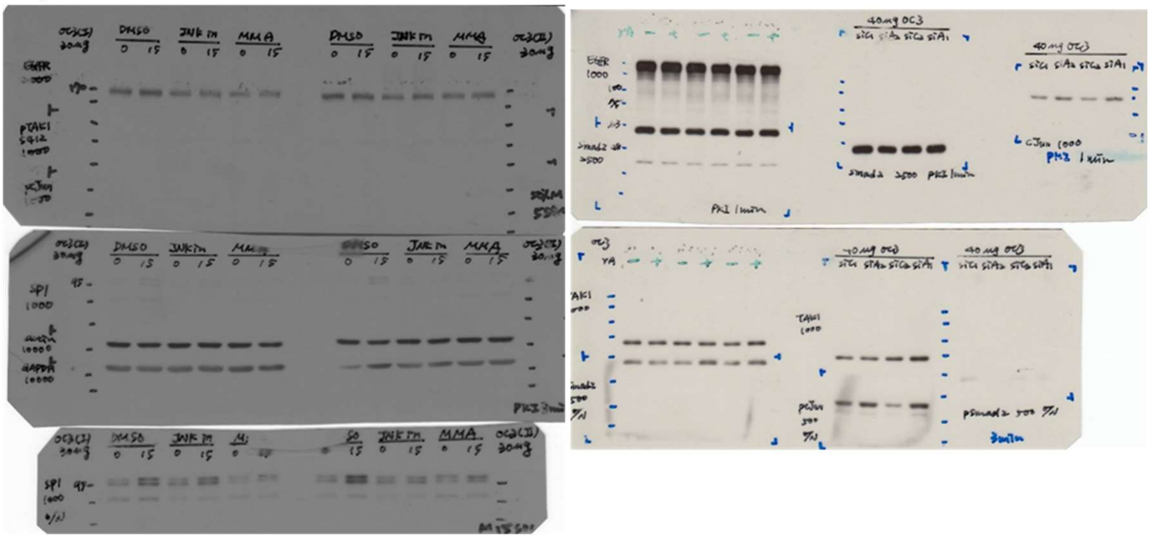

S2a Fig

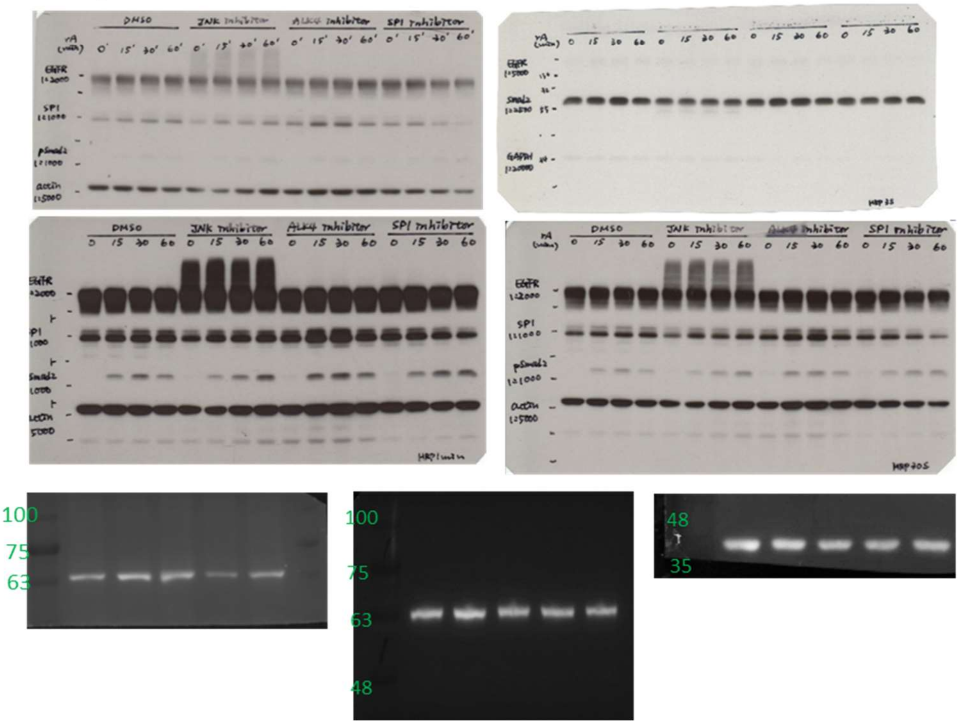

S2b Fig

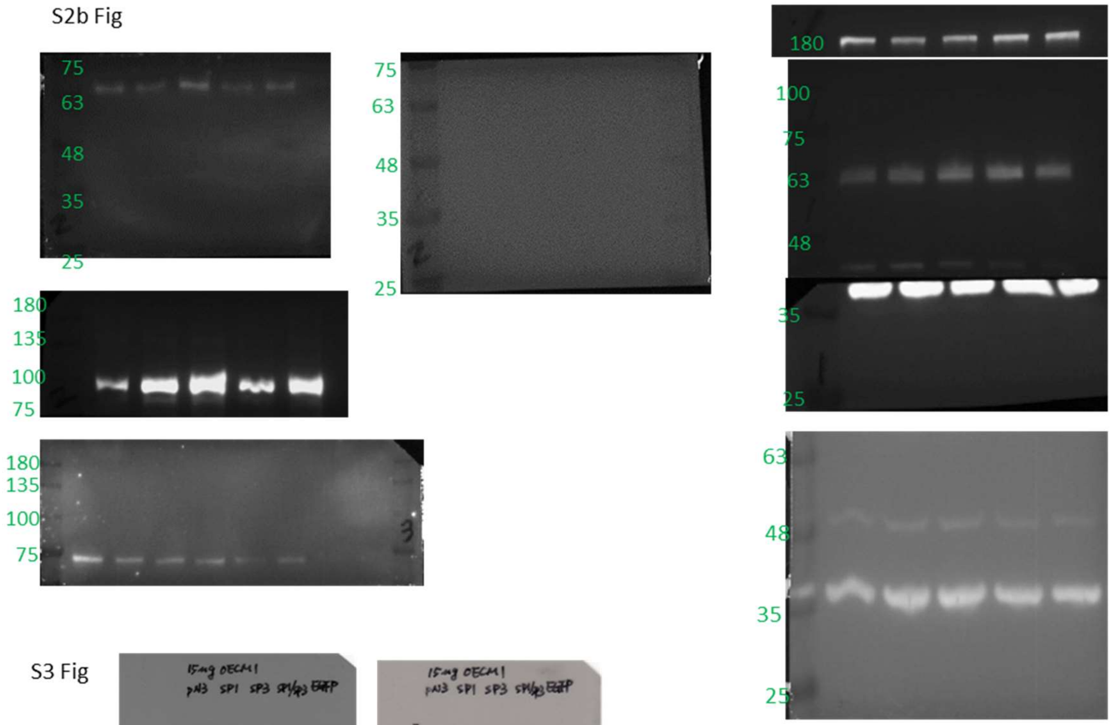

S3 Fig

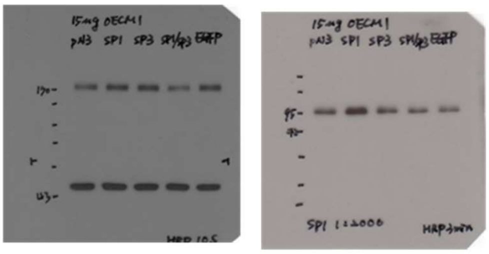

S4 Fig

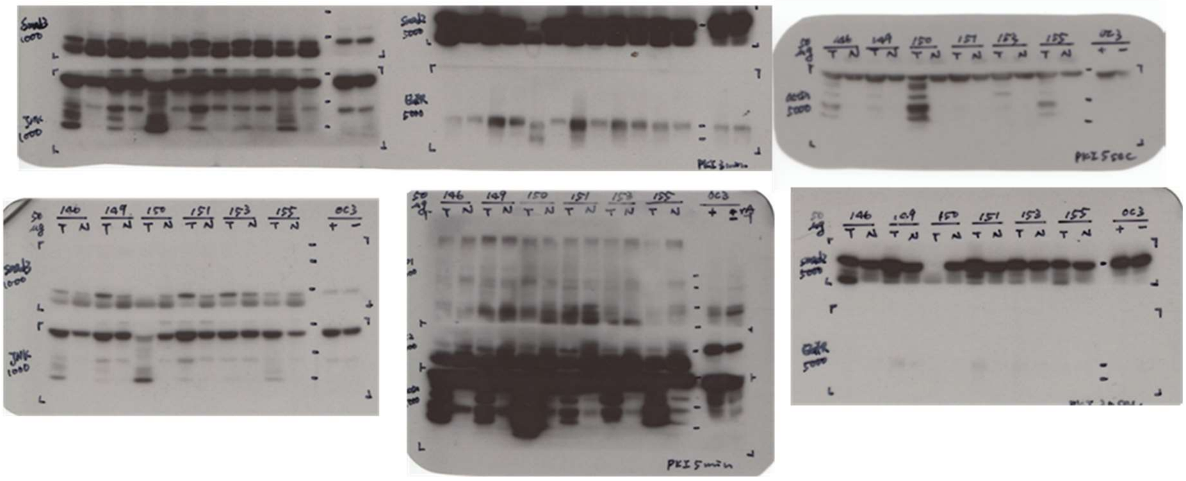

Supplement: Supplementary file 1 — Supplemental information [file 41598_2019_41396_MOESM1_ESM.pdf]
